# Patchy profile sign in RAPID software: a specific marker for intracranial atherosclerotic stenosis in acute ischemic stroke

**DOI:** 10.3389/fneur.2024.1414959

**Published:** 2024-05-30

**Authors:** Lingwen Zhang, Hua Xue, Xiaoqing Bu, Juan Liao, Ge Tang, Yu Chen, Libo Zhao, Deyu Yang, Li Liu, Shudong Liu

**Affiliations:** ^1^Department of Neurology, Yongchuan Hospital of Chongqing Medical University, Chongqing, China; ^2^Chongqing Key Laboratory of Cerebrovascular Disease Research, Yongchuan Hospital of Chongqing Medical University, Chongqing, China; ^3^Department of Epidemiology, School of Public Health, Chongqing Medical University, Chongqing, China; ^4^Department of Health Management, Yongchuan Hospital of Chongqing Medical University, Chongqing, China

**Keywords:** ischemic stroke, intracranial embolism, perfusion, software, atherosclerotic, computed tomography

## Abstract

**Purpose:**

Identifying the etiology of acute ischemic stroke (AIS) before endovascular treatment (EVT) is important but challenging. In CT perfusion imaging processed by perfusion software, we observed a phenomenon called patchy profile sign (PPS), that is, the hypoperfusion morphology in RAPID software is a discontinuous sheet pattern. This phenomenon is predominantly observed in patients diagnosed with intracranial atherosclerotic stenosis (ICAS). The study intends to assess whether the PPS can be used to differentiate ICAS from intracranial embolism.

**Method:**

Patients with AIS due to M1 segment occlusion of the MCA who underwent mechanical thrombectomy were retrospectively enrolled. The receiver operating characteristic (ROC) curve analysis was performed to assess the value of PPS in predicting ICAS. Sensitivity, specificity, positive predictive value (PPV), negative predictive value (NPV), and accuracy of the PPS for prediction of ICAS were calculated.

**Results:**

A total of 51 patients were included in the study. The PPS was observed in 10 of 19 (52.6%) patients with ICAS, and in 2 of 32 (6.3%) patients with intracranial embolism (*p* < 0.001). Interobserver agreement for identifying PPS was excellent (κ = 0.944). The sensitivity, specificity, PPV, NPV, and accuracy of the PPS for predicting ICAS were 52.6, 93.8, 83.3, 76.9, and 78.4%, respectively.

**Conclusion:**

The PPS on RAPID software is an imaging marker with high specificity for ICAS. Larger sample sizes are imperative to validate the findings.

## 1 Introduction

Acute ischemic stroke (AIS) resulting from large vessel occlusion (LVO) stands as a prominent cause of global morbidity and mortality. Mechanical thrombectomy (MT) has emerged as a pivotal intervention, notably improving outcomes in LVO (1–6). Nevertheless, the prognosis of certain patients undergoing EVT for LVO remains suboptimal, primarily attributable to the interval between onset and intervention, surgical procedure duration, and the volume of cerebral ischemia during interventional procedures (7). Intracranial atherosclerotic stenosis (ICAS) and embolic etiologies constitute the primary pathogenesis of LVO, with ICAS prevalent in Asian populations (8, 9). Additionally, ICAS-related LVO presents distinct challenges, including lower recanalization rates and extended procedural durations (10–12). Owing to subsequent platelet aggregation, patients with ICAS frequently encounter residual stenosis and reocclusion during mechanical thrombectomy. In cases of reocclusion, considering rescue treatment, such as balloon and stent angioplasty, is advisable (13–16). Clear delineation of pathogenesis before intervention will aid in formulating a personalized treatment strategy and enhance the procedural workflow. Preoperative differentiation between ICAS and embolic LVO remains imperative, yet effective imaging biomarkers are lacking, warranting further investigation.

Perfusion imaging utilizing artificial intelligence (AI) software has become a primary modality for preoperative MT assessment. This modality automatically performs image postprocessing of CTP imaging system-derived images, accurately identifying and quantifying the infarct core and ischemic penumbra (17, 18). Multiple clinical studies have confirmed that patients identified using the RAPID software based on perfusion imaging may derive benefits from endovascular therapy within an extended time window (19, 20). Parameter of perfusion imaging, such as the hypoperfusion intensity ratio (HIR) is valuable in discerning collateral flow in patients with anterior LVO (21). Both the HIR and cerebral blood volume (CBV) index are associated with underlying ICAS and may function as predictors of ICAS before EVT (22).

In our clinical practice, we have documented the patchy profile sign (PPS), an observed phenomenon in some patients where the hypoperfusion morphology manifests as a non-continuous sheet pattern. Specifically, we have observed that PPS is more prone to manifest in patients with ICAS-associated LVO. We postulated that PPS could serve as a valuable imaging marker for predicting ICAS before EVT. This study aims to objectively ascertain whether the PPS on RAPID software can effectively differentiate between ICAS and intracranial embolism before EVT.

## 2 Materials and methods

The study was approved by the Ethics Committee, and the need for informed consent was waived for the retrospective nature of the study. The procedures of this study adhere to the declaration of Helsinki.

### 2.1 Study participants

**Figure 1:**
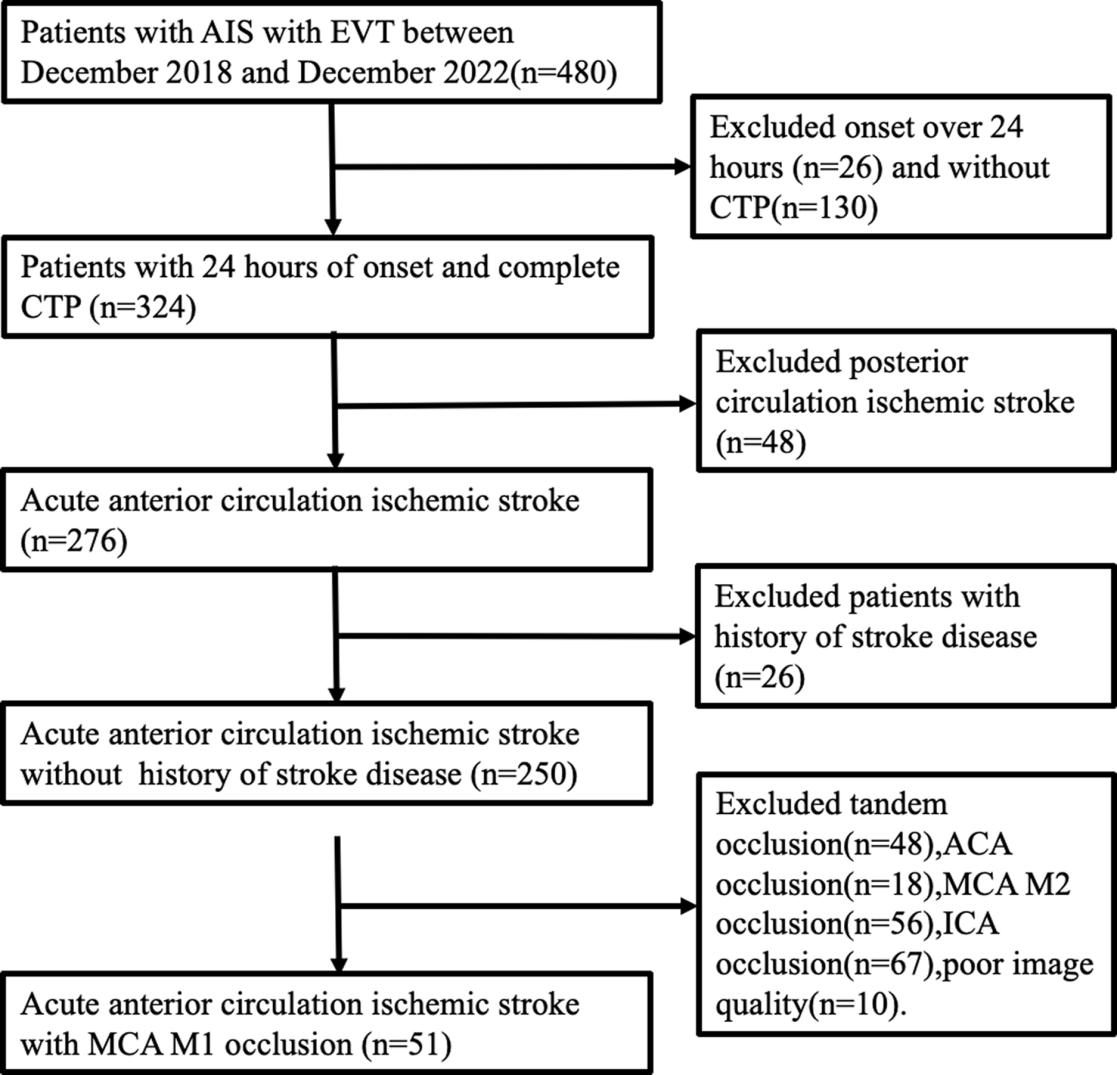
Patient selection process. AIS, acute ischemic stroke; EVT, endovascular treatment; ACA, anterior cerebral artery.

The study retrospectively involved 51 patients selected from our database of consecutive AIS patients who underwent emergency EVT at the Comprehensive Stroke Center from December 2018 to December 2022. Figure 1 depicts the patient screening process. Inclusion criteria comprised individuals aged over 18, admitted within 24 h from symptom onset, undergoing CTP within 24 h of onset, experiencing ischemic stroke due to MCA M1 occlusion, and receiving EVT with successful recanalization (defined as an mTICI grade of 2b-3 or eTICI grade of 2b-3). Exclusion criteria involved individuals with MCA M1 lesions lacking embolism or stenosis, tandem lesions of the MCA, a history of stroke disease, incomplete clinical or imaging data, or poor image quality (concurrent intracranial structural lesions or strenuous activity during image refinement). Collected clinical data of study participants encompassed demographic characteristics (age and gender), stroke risk factors (history of hypertension, diabetes, atrial fibrillation, hyperlipidemia, smoking, and drinking), and clinical characteristics (Intravenous thrombolysis, systolic and diastolic blood pressure, admission random intravenous blood glucose, admission NIHSS, 24-h post-operative NIHSS, admission Glasgow Coma Scale [GCS], ASPECTS, onset to imaging time, onset to puncture time). Data supporting the study can be obtained from the corresponding authors upon reasonable request.

### 2.2 Imaging data

All stroke patients underwent a comprehensive CT scan, consisting of a non-contrast CT, CT angiography of the head and neck, and CT perfusion, using a 256-slice multi-detector CT scanner (Brilliance iCT). Initially, a non-contrast CT scan of the head was performed to rule out intracranial bleeding, followed by a CT angiogram of the head and neck, and subsequent CT perfusion imaging. Whole-brain helical NCCT (120 kVp,100–350 auto-mAs) was performed with 5-mm section thickness. CT perfusion parameters were obtained in a periodic spiral pattern. A high-pressure syringe was utilized to inject 70–90 mL of the contrast agent iopamidol at a flow rate ranging from 4.0 to 6.0 mL/s. Subsequently, the tube was flushed with 30 mL of physiological saline, and the scan commenced with a 5-s delay. The imaging spanned from the foramen magnum to the level above the lateral ventricle, utilizing an 80 mm collimation, tube voltage of 80 kV, and tube current of 100 mA. The perfusion maps and their associated parameters underwent automated analysis using the RAPID software (iSchemaView, Menlo Park, CA; version 5.0.4). The ischemic core was defined as a tissue volume with cerebral blood flow of <30% on CTP imaging. Hypoperfusion was defined as a volume of tissue of Tmax >6 s on CTP. The mismatch ratio was calculated by dividing the ischemic core volume by the lesion volume with Tmax >6 s. The mismatch volume was calculated by subtracting the ischemic core volume from the lesion volume with a Tmax >6 s. HIR was defined as the ratio of the volume of the “Tmax >10 s” lesion divided by the volume of the “Tmax >6 s” lesion. The CBV index was defined as the ratio of the mean CBV within the “Tmax >6 s” lesion in the ipsilateral hemisphere over the mean CBV of the unaffected brain area. We defined HIR ≤ 0.22 and CBV ≥ 0.90 as favorable predictors of atherosclerosis based on previous research (22).

Collateral status on CTA was assessed by a straightforward method, which assesses the backfilling of the soft meningeal arteries in the entire MCA ischemic area compared to the contralateral side, defined as (0, minimal; 1, less than 50%; 2, greater than 50%; 3, filling 100% of the ischemic area) (23).

### 2.3 Operational definitions of ICAS and intracranial embolism

**Figure 2:**
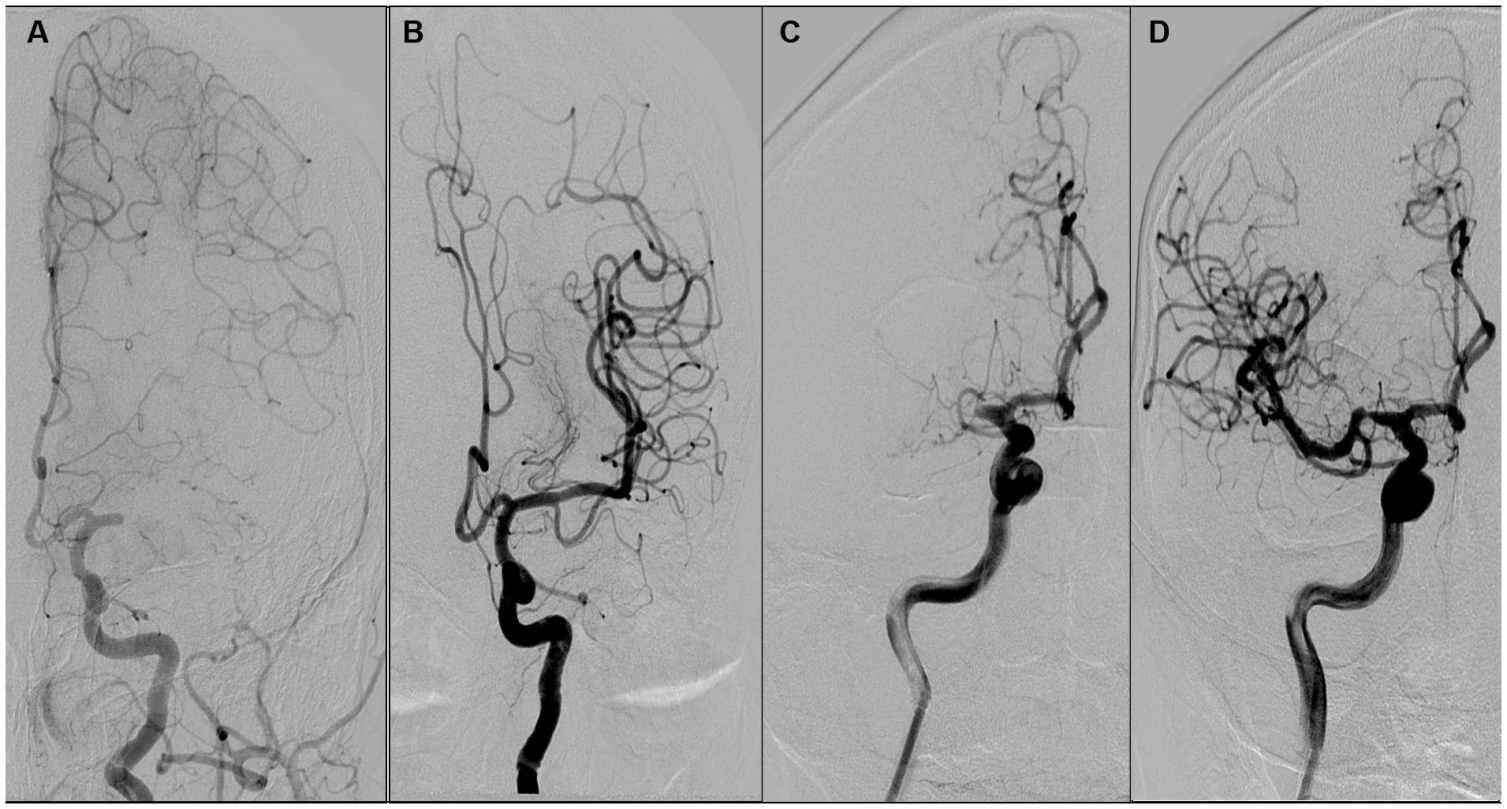
Illustration of the definition of atherosclerosis and embolism. **(A,B)** suggest that the etiology of occlusion is an intracranial embolism, while **(C,D)** suggest that occlusion is due to atherosclerosis. A female patient aged 68 years with occlusion of the left middle cerebral artery M1 **(A)**, which was revascularized after arterial suction thrombectomy **(B)**. A male patient aged 68 years with occlusion of the right middle cerebral artery M1 **(C)**, which was revascularized with antiplatelet therapy and stent thrombectomy **(D)**.

ICAS was differentiated from embolism based on the outcome of angiographic findings and endovascular treatment. ICAS was defined as (1) fixed stenosis ≥70% with either angiographically evident impaired perfusion or evidence of reocclusion following sufficient treatment with a stent retriever, and (2) percutaneous transluminal angioplasty and dual antiplatelet therapy were required to maintain effective recanalization (Figures 2C,D). Iatrogenic dissection or vessel wall injury resulting in stenosis was not classified as an ICAS and was excluded from the study. Embolism was classified as there was no or certain focal stenosis and no tendency for reocclusion after clot retrieval. If CTA within 1 week of surgery confirms complete recanalization of the responsible vessel, it is also considered an embolism (22, 24) (Figures 2A,B).

### 2.4 Definition of the PPS

**Figure 3:**
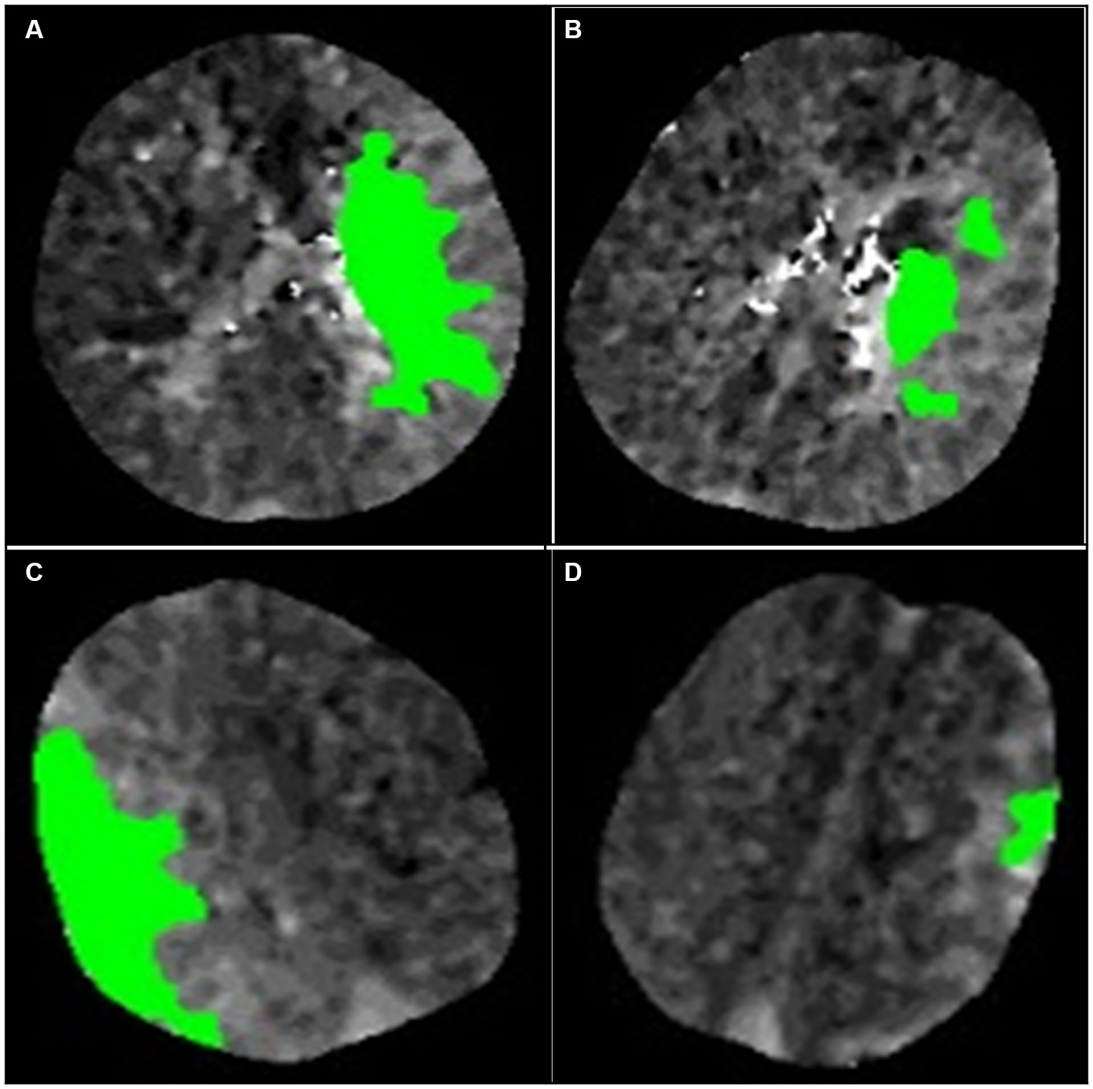
Illustration of the definition of the patchy profile and its mimics. **(A,B)** are considered images with patchy profiles. **(C,D)** are considered patchy profile mimics. **(A)** The image is a complete composition, but the edges of the image are extremely irregular and do not satisfy any definition of geometric morphology. **(B)** The image consists of three patches of similar size. **(C)** The image consists of a complete figure but with a smooth curve on one edge. **(D)** The image consists of a patch of minor area.

The positive PPS was defined as (1) a hypoperfusion region comprising two or more comparable scattered patches or primarily contiguous regions with highly irregular edges that do not conform to any geometric or morphological definition. (Figures 3A,B) (2); the aforementioned images accounted for half or more of all images in the presence of hypoperfusion. Small patches of hypoperfusion images considered individually insignificant were not included in the preceding definition. Similarly, images displaying regular edges on one side were also excluded. (Figures 3C,D).

**Figure 4:**
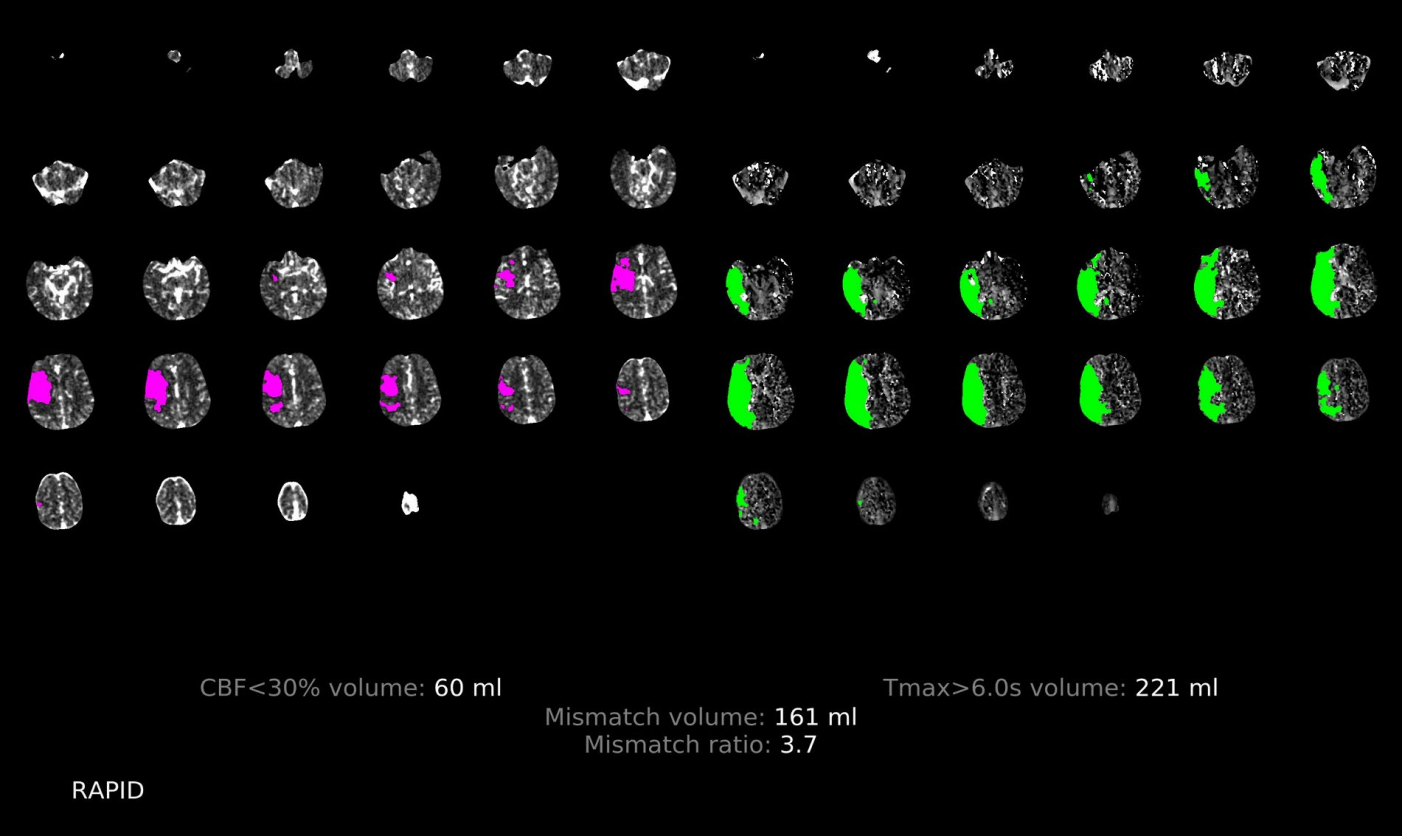
Illustration of the patchy profile sign negative. A male patient aged 68 years with occlusion of the right middle cerebral artery M1, where images with the patchy profile sign accounted for less than half of all images with hypoperfusion.

**Figure 5:**
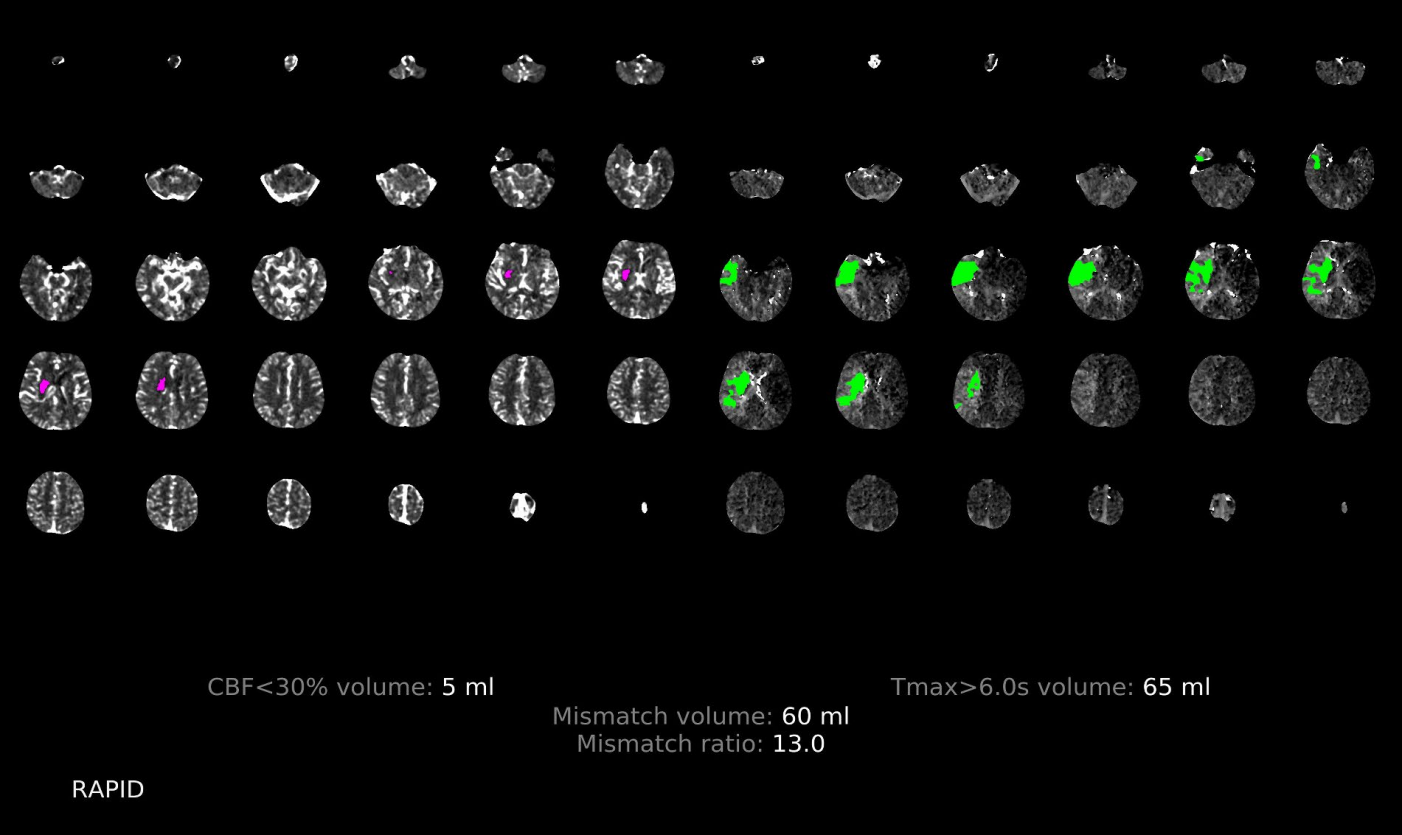
Illustration of the patchy profile sign positive. A male patient aged 66 years with occlusion of the right middle cerebral artery M1, where images with the patchy profile sign accounted for more than half of all images with hypoperfusion.

Both physicians were trained on irregular profile and simulant images by means of photographs, and all perfusion images were concealed from any associated information, independently evaluated by two neurologists each with over 3 years of experience. The two neurologists discussed the controversial findings until a consensus was reached. The imaging features of hypoperfusion in RAPID software with and without PPS are illustrated in Figures 4, 5.

### 2.5 Statistical analysis

Normally distributed data were expressed as mean ± standard deviation (SD), and the student *t* test was used for comparisons between groups. Non-normally distributed data were presented as median (M) with upper and lower quartiles (P25, P75), and the Mann–Whitney *U* test performed the comparison between groups. Categorical variables were expressed as frequencies (percentages, %). The χ^2^ tests and Fisher exact tests were used to analyze categorical variables as appropriate. The consistency of the observer in identifying PPS was examined by the Kappa consistency test, with consistency defined as κ = 0.01 to 0.20, 0.21 to 0.4, 0.41 to 0.6, 0.61 to 0.8 and 0.81 to 0.99 indicating slight, fair, moderate, substantial, and excellent interobserver agreement, respectively. The area under the ROC curve (AUC) was performed to assess the value of HIR, CBV, and PPS in predicting ICAS. Sensitivity, specificity, positive predictive value (PPV), negative predictive value (NPV), and accuracy were calculated to investigate the diagnostic value of PPS for ICAS. All statistical analyses were performed with IBM SPSS Statistics 26.0 (IBM Corporation, Armonk), and *p* < 0.05 was statistically significant.

## 3 Results

### 3.1 Patient characteristics

A total of 480 patients with AIS receiving endovascular treatment were screened, of which 51 met the criteria for inclusion in the analysis. The cause of vascular occlusion was intracranial embolism in 32 patients and ICAS in 19 patients. The PPS was positive in 12 patients and negative in 39 patients.

### 3.2 Patients with and without PPS

The PPS was observed in 12 (23.5%) of the 51 study patients. The consistency of the two observers in identifying PPS was excellent (Kappa test, κ = 0.944). Clinical characteristics and imaging features of patients with and without PPS are displayed in Table 1. Compared to patients without PPS, patients with PPS had smaller ischemic core (0 vs. 15.0, *p* = 0.001), smaller hypoperfusion areas (61.5 vs. 158.8, *p* < 0.001), lower HIR index (0.1 vs. 0.5, *p* < 0.001), smaller mismatch volumes (60.6 vs. 128.4, *p* < 0.001), higher CBV index (0.8 vs. 0.7, *p* = 0.01), lower random venous glucose (5.5 vs. 6.9, *p* = 0.046), and less often hyperlipidemia (0 vs. 35.9%, *p* = 0.02). There were no significant differences in gender, age, smoking, drinking, hypertension, diabetes, atrial fibrillation, systolic blood pressure, diastolic blood pressure, intravenous thrombolysis, admission NIHSS, admission GCS, 24-h post-operative NIHSS, onset to imaging time, onset to puncture time, mismatch ratio, fasting blood glucose, CTA collateral score, and ASPECTS between the two groups (all *p* values >0.05).

**Table 1:** Clinical, Demographic, and Radiological Characteristics of Patients with and without Patchy Profile Sign.

Variables	Patchy profile sign positive (*n* = 12)	Patchy profile sign negative (*n* = 39)	*P* value
Male, *n* (%)	8 (66.7)	20 (51.3)	0.35
Age, years	69.1 ± 12.7	68.9 ± 11.5	0.97
Smoking, *n* (%)	5 (41.7)	11 (28.2)	0.48
Drinking, *n* (%)	4 (33.3)	11 (28.2)	0.73
Hypertension, *n* (%)	7 (58.3)	16 (41.0)	0.29
Diabetes, *n* (%)	2 (16.7)	11 (28.2)	0.71
Hyperlipidemia, *n* (%)	0 (0.0)	14 (35.9)	0.02
Onset to imaging time (min)	210.0 (158.0, 480.0)	240.0 (150.0, 420.0)	0.70
Onset to puncture time (min)	300.0 (240.0, 713.0)	390.0 (270.0, 720.0)	0.52
Atrial fibrillation, *n* (%)	4 (33.3)	18 (46.2)	0.43
Intravenous thrombolysis, *n* (%)	4 (33.3)	9 (23.1)	0.47
Systolic blood pressure, (mmHg)	147.0 ± 15.4	145.0 ± 28.4	0.82
Diastolic blood pressure (mmHg)	84.2 ± 10.5	79.9 ± 16.2	0.39
Random intravenous blood glucose (mmol/L)	5.5 (5.3, 6.9)	6.9 (5.7, 10.8)	**0.046**
Admission NIHSS scores	12.0 (8.0, 19.0)	16.0 (12.0, 21.0)	0.051
24 h NIHSS	8.4 ± 7.4	11.2 ± 7.1	0.25
GCS	15.0 (12.0, 15.0)	13.0 (10.0, 15.0)	0.06
CBF < 30%, (mL)	0.0 (0.0, 0.0)	15.00 (0.0, 48.0)	**0.001**
Tmax>6 s, (mL)	61.5 ± 38.2	158.8 ± 50.2	**<0.001**
Mismatch ratio^#^	13.9(13.0, 14.7)	5.5 (2.8, 12.0)	0.13
HIR	0.1 (0, 0.2)	0.5 (0.4, 0.6)	**<0.001**
CBV	0.8 (0.7, 1.0)	0.7 (0.6, 0.8)	**0.01**
CTA collateral score			0.07
Good (3)	6 (50. 0)	7 (17. 9)	
Intermediate (2)	5 (41. 7)	23 (60. 0)	
Poor (0–1)	1 (8.3)	9 (23. 1)	
ASPECTS	8.5 (7.0,9.8)	7.0 (6.0, 9.0)	0.10
Mismatch volume, (mL)*	60.6 ± 37.8	128.4 ± 44.3	**<0.001**

^#^Mismatch ratio is defined as Tmax > 6 s divided by CBF < 30%. *Mismatch volume refers to the regions where Tmax > 6 s does not match CBF < 30%. GCS, Glasgow Coma Scale; CBV, cerebral blood volume; HIR, hypoperfusion intensity ratio.

### 3.3 Patients with ICAS vs. patients with intracranial embolism

Patients with ICAS were diagnosed in 19 (37.25%) of the 51 patients. The clinical characteristics and imaging features of patients with ICAS and intracranial embolism are shown in Table 2. Patients with ICAS were more likely to be smoking (52.6% vs. 18.8%, *p* = 0.01), more often than males (78.9% vs. 40.6%, *p* = 0.008), less likely to have atrial fibrillation (10.5% vs. 62.5%, *p* < 0.001) and more often to have positive PPS (52.6% vs. 6.3%, *p* < 0.001). The volume of the ischemic core (0 vs. 15.0, *p* = 0.002), the volume of hypoperfusion (105.3 vs. 154.1, *p* = 0.006), and the HIR index (0.3 vs. 0.5, *p* = 0.001), the admission NIHSS score (12.0 vs. 17.0, *p* = 0.003) were lower in the ICAS group than those in the intracranial embolism group. The CBV index (0.8 vs. 0.7, *p* = 0.02) and ASPECTS (9.0 vs. 7.0, *p* = 0.005) were higher in the ICAS group than in the intracranial embolism group. There were no significant differences in age, drinking, hypertension, diabetes, hyperlipidemia, random venous glucose, intravenous thrombolysis, systolic and diastolic blood pressure, 24-h post-operative NIHSS, admission GCS, onset to imaging time, onset to puncture time, mismatch ratio, mismatch volume, CTA collateral score between the two groups (all *p* values >0.05).

**Table 2:** Clinical, demographic, and radiological characteristics of patients with ICAS and intracranial embolism.

Variables	The ICAS (*n* = 19)	The IE (*n* = 32)	*P* value
Male, *n* (%)	15 (78.9)	13 (40.6)	**0.008**
Age, years	65.5 ± 11.6	71.0 ± 11.4	0.10
Smoking, *n* (%)	10 (52.6)	6 (18.8)	**0.01**
Drinking, *n* (%)	8 (42.1)	7 (21.9)	0.13
Hypertension, *n* (%)	11 (57.9)	12 (37.5)	0.16
Diabetes, *n* (%)	5 (26.3)	8 (25.0)	1.00
Hyperlipidemia, *n* (%)	3 (15.8)	11 (34.4)	0.15
Onset to imaging time (min)	240.0 (180.0, 600.0)	240.0 (127.5, 382.5)	0.12
Onset to puncture time (min)	390.0 (280.0, 990.0)	360.0 (232.5, 585.0)	0.14
Atrial fibrillation, *n* (%)	2 (10.5)	20 (62.5)	**<0.001**
Intravenous thrombolysis, *n* (%)	5 (26.3)	8 (25.0)	1.00
Systolic blood pressure, (mmHg)	154.2 ± 26.8	140.3 ± 24.2	0.06
Diastolic blood pressure, (mmHg)	85.0 ± 18.6	78.5 ± 12.3	0.14
Random intravenous blood glucose (mmol/L)	5.9 (5.3, 9.2)	6.7 (5.6, 10.7)	0.28
Admission NIHSS scores	12.0 (8.0, 19.0)	17.0 (13.0, 21.8)	**0.003**
24 h NIHSS	9.2 ± 6.5	11.3 ± 7.6	0.30
GCS	15.0 (11.0, 15.0)	12.5 (10.0, 15.0)	0.12
CBF < 30%, (mL)	0.0 (0.0, 5.0)	15.0 (0.0, 6.0)	**0.002**
Tmax>6 s, (mL)	105.3 ± 67.2	154.1 ± 53.6	**0.006**
Mismatch ratio^#^	12.7 (6.7, 18.2)	5.2 (2.6, 11.7)	0.05
HIR	0.3 (0.0, 0.4)	0.5 (0.4, 0.6)	**0.001**
CBV	0.8 (0.8, 0.9)	0.7 (0.6, 0.8)	**0.02**
CTA collateral score			0.71
Good (3)	6 (31.6)	7 (21.9)	
Intermediate (2)	10 (52.6)	18 (56.3)	
Poor (0–1)	3 (15.8)	7 (21.9)	
ASPECTS	9.0 (7.0, 10.0)	7.0 (6.0, 8.8)	**0.005**
Mismatch volume, (mL)*	100.7 ± 64.6	119.4 ± 41.5	0.27
Patchy Profile Sign	10 (52.6)	2 (6.3)	**<0.001**

^#^Mismatch ratio is defined as Tmax > 6 s divided by CBF < 30%. *Mismatch volume refers to the regions where Tmax > 6 s does not match CBF < 30%. ICAS = intracranial atherosclerotic stenosis; CBV = cerebral blood volume; GCS = Glasgow Coma Scale; HIR = hypoperfusion intensity ratio.

### 3.4 Diagnostic performance of the PPS

**Figure 6:**
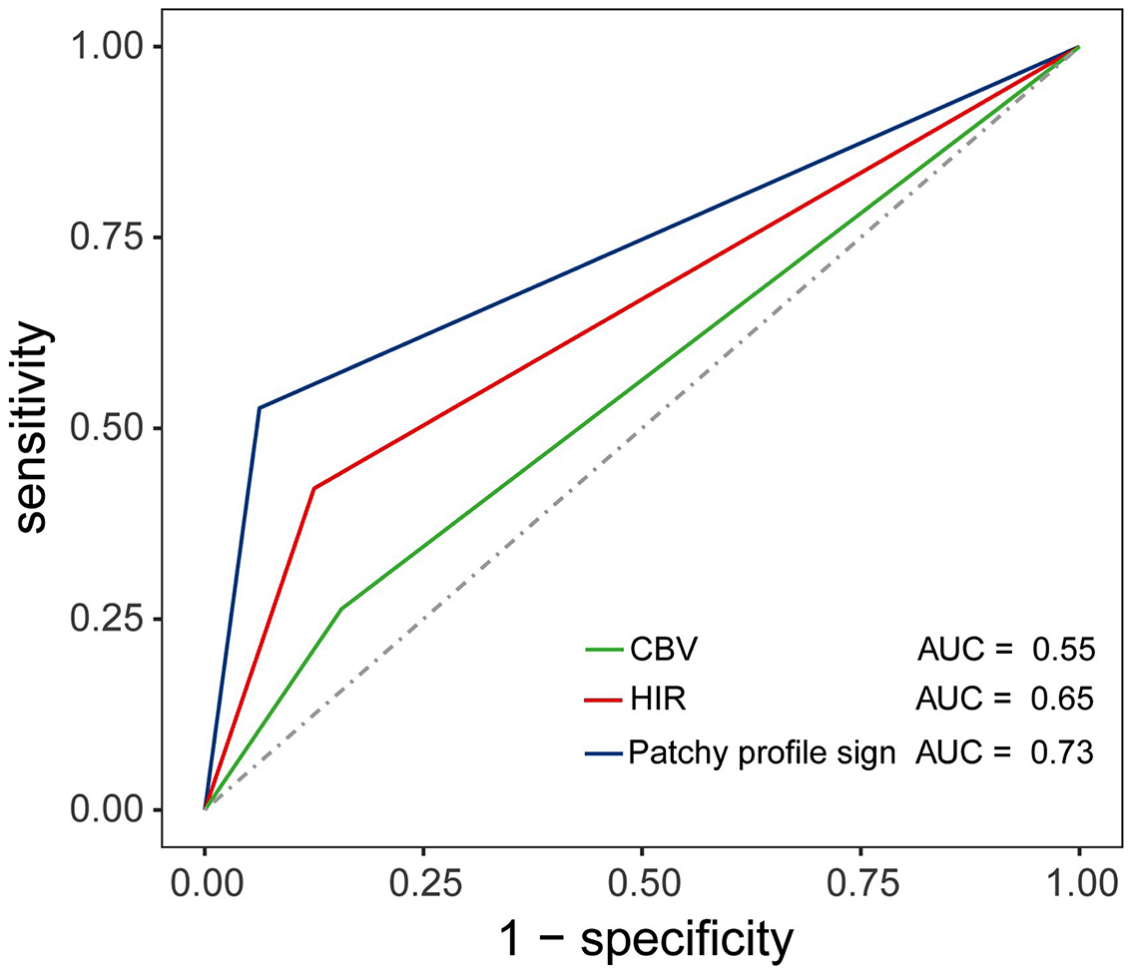
Illustration of the receiver operating characteristic (ROC) curve depicting the diagnostic value of HIR, CBV, and the patchy profile sign in predicting ICAS. The area under the curve (AUC) confirmed the superior value of the patchy profile sign in predicting ICAS (AUC, 0.73; *p* = 0.003) compared to HIR (AUC, 0.65; *p* = 0.08) and CBV (AUC, 0.55; *p* = 0.53).

The PPS was positive in 10 of the 19 patients in the ICAS group but in only 2 of the 32 patients in the intracranial embolism group (52.6% vs. 6.3%, respectively, *p* < 0.001). The sensitivity, specificity, PPV, NPV, and accuracy of PPS for detecting ICAS were 52.6, 93.8, 83.3, 76.9, and 78.4%. The PPS showed a better performance in predicting ICAS (AUC, 0.73; 95% CI: 0.58, 0.89; *p* = 0.003) over HIR ≤ 0.22 (AUC, 0.65; 95% CI: 0.48, 0.81; *p* = 0.08) and CBV ≥ 0.90 (AUC, 0.55; 95% CI: 0.39, 0.72; *p* = 0.53). The ROC curves are shown in Figure 6.

## 4 Discussion

In the study, an effortless and time-saving imaging technique was discovered, which facilitates the differentiation between intracranial atherosclerotic stenosis and intracranial embolism in acute anterior circulation stroke patients with MCA M1. The study found that the presence of PPS is more likely in patients with ICAS-related LVO. The existing literature on the prognostic capacity of AI software images for stroke etiology is limited. This study could contribute to facilitating further research on AI software imaging across a diverse range of clinical practitioners.

### 4.1 Potential mechanisms underlying PPS

The PPS may arise because the collateral circulation of patients with atherosclerotic stenosis is better than that of patients with embolism due to the chronic and usually slow progression, allowing sufficient time for opening and formation of collateral circulation (24–26). While the CTA collateral score did not reach statistical significance in this study, there was an observable proportional disparity between the positive and negative groups (22). The proportion of patients with good collateral circulation was significantly higher in the PPS-positive group compared with the PPS-negative group, whereas the proportion of patients with poor collateral circulation was significantly lower. Such findings potentially stem from the limited sample size inherent to our study and disparities in the methodologies employed for collateral circulation assessment. Consequently, a comprehensive large sample study is warranted to validate and substantiate our hypothesis. Chronic cerebral hypoperfusion may promote the formation of intracranial collateral arteries, as demonstrated in an animal model of bilateral carotid artery occlusion (27). Furthermore, the establishment of intracranial collateral circulation is related to the severity and velocity of cerebral vascular stenosis (28). Additionally, a previous study demonstrated that chronic cerebrovascular stenosis induces prolonged cerebral hypoperfusion, inducing a hypoxic-tolerant state in brain tissue. This results in an increased concentration of vascular growth factors, promoting the establishment of collateral circulation, and providing protection against cerebral ischemia (29). Furthermore, it was found that some patients who underwent endovascular treatment and achieved complete recanalization still experienced considerable hypoperfusion on rapid perfusion analysis. The mechanism behind this phenomenon remains unclear and may be related to inadequate microcirculatory reperfusion (30, 31). ICAS-related LVO with a more robust collateral circulation may encounter improved microcirculatory reperfusion after occlusion, leading to patchy areas of hypoperfusion. In contrast, embolization-related LVO may experience inadequate microcirculatory reperfusion due to the rapid occlusion, frequently exhibiting areas of hypoperfusion nearly identical to the region supplied by the responsible vessel. Our hypothesis posits that the presence of PPS in the hypoperfusion region may be attributed to the superior microcirculatory reperfusion in ICAS-related LVO compared to embolization-related LVO. Naturally, the confirmation of specific pathophysiological mechanisms requires further studies.

### 4.2 Imaging characteristics of ICAS-related LVO vs. embolism-related LVO

The study found that ICAS-related LVO had smaller areas of hypoperfusion compared to embolization-related LVO, which is consistent with prior investigations (22, 32). Contrary to embolization-related LVO, EVT for ICAS-related LVO is associated with a higher incidence of intraoperative reocclusion, an extended operating time, elevated mortality rates, and reduced revascularization rates (33). In the management of ICAS-related LVO, interventions such as stenting, angioplasty, or a combination of these techniques are typically employed to achieve successful revascularization. Accurate identification of potential ICAS and the development of an optimal strategy for ICAS-related LVO ensure more effective recanalization of occluded vessels and a favorable prognosis for patients with this condition (34, 35). In this study, patients with ICAS were more likely to be smokers but exhibited lower rates of atrial fibrillation, aligning with the findings of a separate study (24). The results of this study indicate that patients with ICAS, in comparison to those with intracranial embolism, manifested a lower NIHSS score, potentially attributed to superior collateral circulation in ICAS patients (26). In addition, the hypoperfusion volume, ischemic core volume, mismatch volume and HIR index in patients with intracranial embolism surpassed those in ICAS patients, while the CBV index in the embolism group was lower than that in the ICAS group. This finding aligns with previous studies (22, 24), and may be associated with the sudden onset, insufficient collateral circulation, and the presence of a large thrombus in patients with intracranial embolism (36).

### 4.3 Challenges in identifying etiology of AIS in emergency settings

It can be challenging to differentiate accurately between these two causes in clinical settings, particularly during emergency scenarios (37). The following imaging techniques are considered beneficial in assessing the nature of the lesions. High-resolution vessel wall MRI could help to identify ICAS (36, 38), while MRI examinations require significant time, patient cooperation, and are impractical for preoperatively assessing emergency surgery candidates. Kim et al. reported that patients positive for hyperdense middle cerebral artery sign (HMCAS) had a higher incidence of intracranial embolism (67.8% vs. 48.9%, *p* = 0.005), whereas HMCAS-negative patients exhibited a higher incidence of ICAS (31.9% vs. 12.7%, *p* = 0.001) (39). However, several studies have demonstrated that HMCAS does not exhibit a significant correlation with the etiology of AIS (40, 41). Currently, identifying the etiology of AIS using HMCAS still requires extensive observational and demonstrative research. Furthermore, ICAS can be identified by the microcatheter first-pass effect observed during digital subtraction angiography (DSA) owing to the low burden of fresh thrombosis (14). However, the inability to clarify the etiology of the disease before surgery limits the promotion of clinical applications. A recent study found that a Tmax ratio of >10 s/>6 s could predict ICAS-related LVO with or without embolic sources before EVT (42). However, the study did not provide a specific cut-off value, and some patients could not calculate the ratio due to a Tmax >6 s value of zero. Therefore, it is necessary to identify more effective and simpler imaging signs to differentiate ICAS-related from embolization-related LVO. A recent study suggested that HIR ≤ 0.22 (AUC, 0.85; 95% CI: 0.75, 0.96) and CBV ≥ 0.90 (AUC = 0.92, 95% CI: 0.81, 0.98) could serve as valid predictive biomarkers for ICAS (22). In this study, we dichotomized the two indices based on the cut-off values and compared them with the PPS. The analysis revealed that PPS had a higher AUC value for predicting atherosclerosis compared with HIR and CBV. Both HIR and CBV parameters are automatically calculated by software based on perfusion images and are currently used to reflect collateral circulation. A recent investigation has affirmed a significant association between favorable HIR and atherosclerosis (43). Importantly, in our current study, we observed the presence of the PPS in the perfusion images, suggesting a potential shared etiological mechanism with the two indicators.

### 4.4 Advantages and limitations of the study

Imaging signs such as thrombus imaging, diffusion-weighted imaging, vascular calcification, and collateral circulation are primarily obtained through catheter angiography or thrombectomy, rather than during routine CT scans (44, 45). MRI scanning can also yield some signs, but it necessitates that the patient keeps their heads motionless throughout the examination period. Recognizing these signs requires the expertise of specialized neurologists and imaging physicians, thus limiting their clinical application. A significant advantage of this research is the introduction of a novel imaging approach that can be easily detected in clinical environments. However, our study has some limitations. First, the study was retrospective with a small sample size, highlighting the need for additional prospective studies with larger sample sizes. Second, this study exclusively included patients with occlusion of the MCA M1. Subgroups were identified through imaging, and patients with persistent vascular occlusion were excluded, leaving uncertainty about whether similar conditions exist in patients with occlusion of other vessels. Third, while congestive heart failure in some patients may lead to decreased cerebral perfusion, it remains unclear whether these patients exhibit differences in AI software perfusion images. Consequently, such patients were not excluded from the present study, potentially introducing selection bias into the results. Furthermore, the imaging analysis in this study was conducted using the RAPID software, which might potentially limit its widespread applicability. Several software options are available for perfusion calculations, and further investigation is necessary to determine whether alternative software can produce comparable outcomes. Further research is required for the differential analysis of imaging features in patients with AIS of various etiologies. This will contribute to a more comprehensive assessment of AIS etiology, guiding the selection of clinical treatment options.

## 5 Conclusion

In conclusion, this study introduces a novel perfusion image sign associated with ICAS. The PPS may function as a specific imaging marker for the identification of ICAS and could potentially guide subsequent endovascular revascularization therapy. Further confirmation through prospective studies with larger sample sizes is necessary to validate the findings of this study.

## Data availability statement

The raw data supporting the conclusions of this article will be made available by the authors, without undue reservation.

## Ethics statement

The studies involving humans were approved by Yongchuan Hospital of Chongqing Medical University Ethics Committee. The studies were conducted in accordance with the local legislation and institutional requirements. The ethics committee/institutional review board waived the requirement of written informed consent for participation from the participants or the participants’ legal guardians/next of kin because of the retrospective nature of the study.

## Author contributions

LinZ: Conceptualization, Data curation, Formal analysis, Writing – original draft. HX: Data curation, Writing – original draft. XB: Data curation, Writing – review & editing. JL: Methodology, Writing – review & editing. GT: Data curation, Writing – original draft. YC: Project administration, Writing – original draft. LibZ: Project administration, Writing – review & editing. DY: Conceptualization, Writing – review & editing. LL: Project administration, Supervision, Writing – review & editing. SL: Conceptualization, Supervision, Writing – review & editing.
